# Drug repurposing in cancer neuroscience: From the viewpoint of the autophagy-mediated innervated niche

**DOI:** 10.3389/fphar.2022.990665

**Published:** 2022-08-29

**Authors:** Jiayan Shi, Jia Xu, Yang Li, Bowen Li, Hui Ming, Edouard C. Nice, Canhua Huang, Qifu Li, Chuang Wang

**Affiliations:** ^1^ State Key Laboratory of Biotherapy and Cancer Center, West China Hospital, and West China School of Basic Medical Sciences and Forensic Medicine, Sichuan University and Collaborative Innovation Center for Biotherapy, Chengdu, China; ^2^ Department of Pharmacology, Provincial Key Laboratory of Pathophysiology, Ningbo University School of Medicine, Ningbo, China; ^3^ Department of Biochemistry and Molecular Biology, Monash University, Melbourne, VIC, Australia; ^4^ Department of Neurology and Key Laboratory of Brain Science Research and Transformation in Tropical Environment of Hainan Province, The First Affiliated Hospital, Hainan Medical University, Haikou, China

**Keywords:** cancer neuroscience, innervated niche, autophagy, drug repurposing, cancer treatment

## Abstract

Based on the bidirectional interactions between neurology and cancer science, the burgeoning field “cancer neuroscience” has been proposed. An important node in the communications between nerves and cancer is the innervated niche, which has physical contact with the cancer parenchyma or nerve located in the proximity of the tumor. In the innervated niche, autophagy has recently been reported to be a double-edged sword that plays a significant role in maintaining homeostasis. Therefore, regulating the innervated niche by targeting the autophagy pathway may represent a novel therapeutic strategy for cancer treatment. Drug repurposing has received considerable attention for its advantages in cost-effectiveness and safety. The utilization of existing drugs that potentially regulate the innervated niche via the autophagy pathway is therefore a promising pharmacological approach for clinical practice and treatment selection in cancer neuroscience. Herein, we present the cancer neuroscience landscape with an emphasis on the crosstalk between the innervated niche and autophagy, while also summarizing the underlying mechanisms of candidate drugs in modulating the autophagy pathway. This review provides a strong rationale for drug repurposing in cancer treatment from the viewpoint of the autophagy-mediated innervated niche.

## Introduction

The first indication of the critically modulating effect of innervation on tumors came from the observation of a process termed perineural invasion (Seefeld and Bargen, 1943). Perineural invasion, a phenomenon in which cancer cells can grow around and eventually invade existing nerves, has been observed in various cancers and is associated with poorer prognosis and low survival rates (Ayala et al., 2001; Liebig et al., 2009; Chen et al., 2019a). Beyond perineural spread, there is an emerging awareness that bidirectional interactions between nerves and cancer cells critically regulate tumor initiation and progression (Magnon et al., 2013; Mauffrey et al., 2019; Venkatesh et al., 2019; Hutchings et al., 2020). Accordingly, Monje and other scientists established a new field called “cancer neuroscience” in 2020 (Monje et al., 2020). Cancer neuroscience focuses on the mechanisms of electrochemical interactions, paracrine interactions, systemic neural-cancer interactions, and cancer-therapy effects on the nervous system to better investigate the close relationship between neurology and cancer science (Monje et al., 2020). A critical node in the communications between nerves and cancer is the innervated niche, which contains the sympathetic nerve, parasympathetic nerve, or sensory nerve (Jin and Jin, 2020). It is characterized by close crosstalk among nerves, cancer cells, and non-malignant cells mediated by acellular components such as nerve-derived neurotransmitters or neuropeptides (Jin and Jin, 2020). The innervated niche is also referred to as the “perineural niche” or “neural regulation in the tumor microenvironment (TME)” in some studies (Chen et al., 2019a; Restaino and Vermeer, 2022). The discovery of the innervated niche as an emerging specialized microenvironment has brought exciting new perspectives to the studies of bidirectional neural-cancer interactions (Zahalka and Frenette, 2020).

In the innervated niche, autophagy has recently been reported as a double-edged sword that plays a significant role (Wang et al., 2018; Stavoe and Holzbaur, 2019; Cai and Ganesan, 2022). Autophagy is a conserved cellular self-degradation process that is important for balancing energy sources at critical times during neuronal development and nutrient stress (Mizushima et al., 2008; Morishita and Mizushima, 2019). Furthermore, autophagy plays a housekeeping role in the innervated niche by removing misfolded or aggregated proteins, clearing damaged organelles, and eliminating intracellular pathogens to maintain homeostasis (Choi et al., 2018; Griffey and Yamamoto, 2022). Apart from this, lethal autophagy leads directly to the death of neurons and glial cells, which contribute to the neuropathy of the innervated niche (Ngwenya and Danzer, 2018; Stavoe and Holzbaur, 2019). As more is known about autophagy in the innervated niche, treatment strategies can be developed to regulate autophagy-related molecules contributing to the innervated niche networks. In this context, changing the innervated niche by regulating the autophagy pathway has emerged as a novel therapeutic opportunity for cancer treatment and has emerged as a novel window for drug repurposing.

Following on from a block-buster era for drug discovery, repurposing old drugs to treat new indications is increasingly becoming an attractive proposition because it is a time-saving and cost-efficient method with high success rates (Pushpakom et al., 2019; Roessler et al., 2021). With a deeper understanding of the hallmarks of cancer and the development of various computer-aided approaches, drug repurposing has the promise to rapidly improve our arsenal of anticancer drugs (Hanahan and Weinberg, 2011; Gonzalez-Fierro and Dueñas-González, 2021; Kirtonia et al., 2021; Mottini et al., 2021). To date, a huge number of preclinical trials have shown that multiple noncancer drugs (antipsychotics, cardiovascular, etc.) have demonstrated off-label antitumor effects (Regulska et al., 2019; Shaw et al., 2021). Repurposing drugs such as β-adrenergic antagonists based on the autophagy-mediated innervated niche is a promising pharmacological approach in cancer neuroscience (Demir et al., 2021; Shaw et al., 2021; Shi et al., 2022). Accordingly, we will mainly focus on the anticancer activity of existing drugs that were not initially intended for cancer therapy from the viewpoint of the autophagy-mediated innervated niche. This review should enable researchers to have a deeper understanding of drug repurposing based on the innervated niche and thus expedite the translation of new anticancer drugs into the clinic.

## Nerve dependence across cancer types

Recent discoveries around cancer neuroscience provides evidence that high intratumoral nerve density is correlated with poor survival and high metastasis across multiple cancer types (Reavis et al., 2020). In hematological cancers, the sympathetic nervous system innervates hematopoietic differentiation and the egress of hematopoietic stem cells from bone marrow (Katayama et al., 2006; Cole et al., 2015). In addition, in head and neck cancer, Amit *et al.* found that tumor-associated neurons are reprogrammed toward an adrenergic phenotype that can stimulate tumor progression and are a potential target for anticancer therapy (Amit et al., 2020). Moreover, a retrospective analysis of breast cancer specimens revealed that increased sympathetic nerve density in tumors was associated with poor clinical outcomes (Kamiya et al., 2019).

Since primary brain tumors are closely related to, or even originate from, neurons, their innervated niche is distinct from that in tumors of other organs. Jin *et al.* proposed that the innervated niche could be categorized into two main categories: intracranial and extracranial innervated niches (Jin and Jin, 2020). Research on the intracranial innervated niches has focused on primary brain tumors that dominate, including glioblastoma, schwannoma, astrocytoma, as well as brain metastases. Venkatesh *et al.* indicated the critical role of active neurons in the brain tumor microenvironment and identified neuroligin-3 (NLGN3) as the leading candidate to promote neuronal activity-regulated cancer growth (Venkatesh et al., 2015). Specifically, active neurons promote the growth of high-grade gliomas via the NLGN3 induced PI3K-mTOR pathway. Subsequently, Venkatesh *et al.* further found that neuron and glioma interactions include electrical and synaptic integration (Venkatesh et al., 2019). Neuronal activity evokes non-synaptic activity-dependent potassium currents, which can be amplified by gap junction-mediated tumor interconnections. Additionally, functional neurogliomal synapses produce postsynaptic currents that are mediated by glutamate receptors of the AMPA subtype (Venkataramani et al., 2019). In astrocytoma, many tumor cells extend ultralong membrane protrusions and use these distinct tumor microtubes through growth-associated protein-43 as routes for enhancing brain invasion (Osswald et al., 2015). As the most common brain tumor type, brain metastasis is a frequent neurological complication of cancer (Gállego Pérez-Larraya and Hildebrand, 2014). Approximately 20–40% of cancer patients with primary extracranial cancer will develop brain metastases during the course of their disease, and the incidence of brain metastasis is even higher than that of primary brain tumors (Maher et al., 2009; Lamba et al., 2017). Similarly, emerging evidence indicates that brain metastases also interact closely with the innervated niche (Zeng et al., 2019; Kleffman et al., 2022). Breast-to-brain metastasis (B2BM) is a common and disruptive form of cancer, and B2BM cells can participate in a neuronal signaling pathway that involves activation by glutamate ligands of N-methyl-d-aspartate receptors (NMDARs) (Zeng et al., 2019). This neuronal signaling pathway is key in model systems of metastatic colonization of the brain and is associated with poor prognosis. Furthermore, the formation of pseudo-tripartite synapses between cancer cells and glutamatergic neurons is stimulated such that the reprogrammed innervated niche becomes more favorable for cancer survival, providing a rationale for brain metastasis. In summary, these intriguing findings indicate that the innervated niche has a bidirectional neural regulation of cancer, allowing for a deeper understanding of antitumor strategies based on the innervated niche.

## Bidirectional interaction between the innervated niche and tumor

**FIGURE 1:**
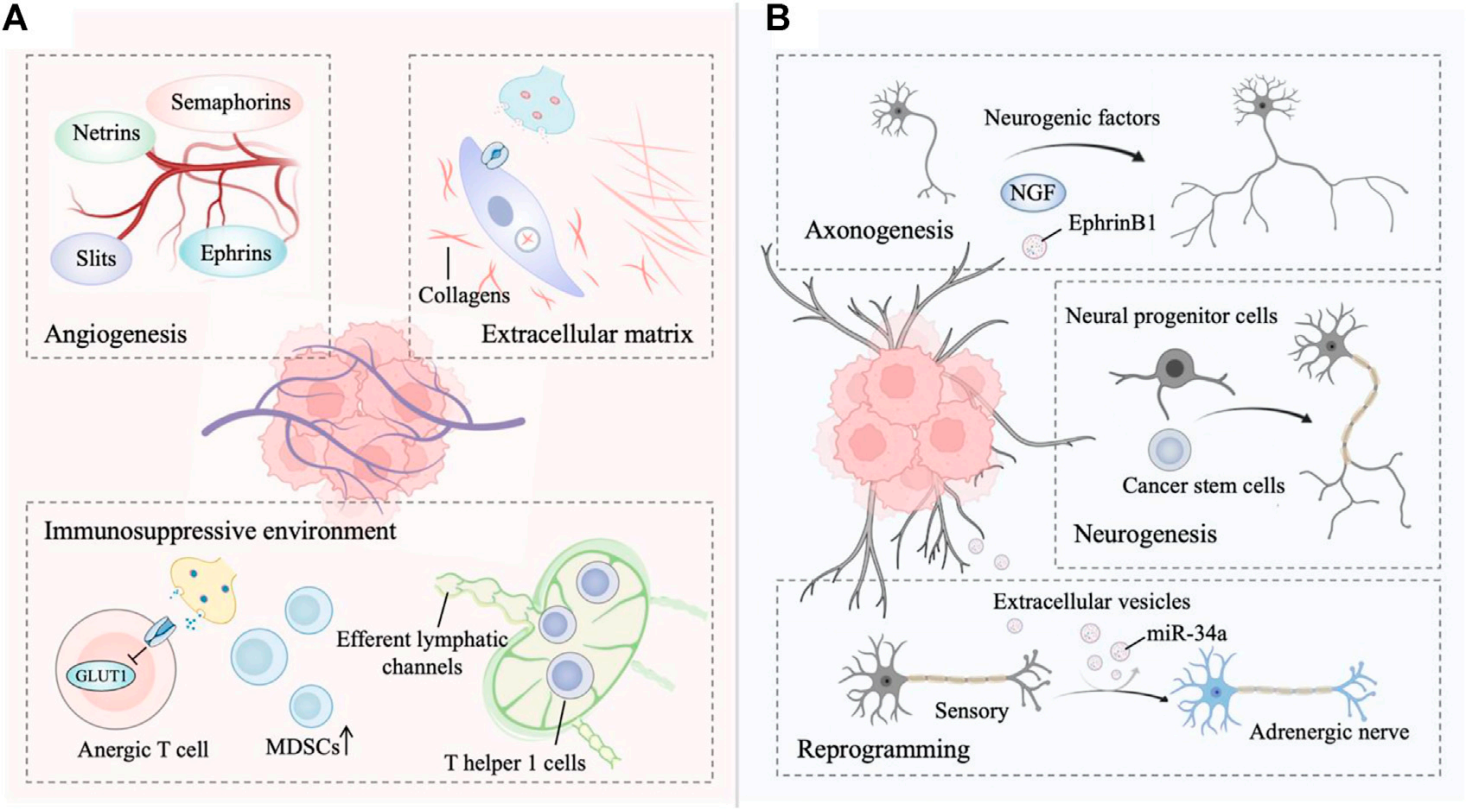
**(A)** The innervated niche regulates tumor progression. Signaling from the innervated niche stimulates angiogenesis by neuronal guidance factors. The innervated niche-regulated extracellular matrix (ECM) is a determinant in cancer dissemination. Adrenergic signaling contributes to a multifaceted immunosuppressive environment. **(B)** Tumors drives innervated niche alteration. Tumors secrete multiple neurogenic factors to promote axonogenesis. Tumors communicate with distant tissues and organs to recruit neural progenitor cells and cancer stem cells for *de novo* neurogenesis. Tumor-secreted factors transform a sensory nerve into an adrenergic nerve to reprogram the innervated niche. This figure was created using BioRender.

The main innervation of cancer cells or non-malignant cells relies on the release of nerve-derived neurotransmitters or neuropeptides, such as dopamine, catecholamine, and acetylcholine (Jin and Jin, 2020). Moreover, nerves secrete a series of membrane-anchored proteins, matrix metalloproteinases, chemokines and non-coding RNAs, that can be delivered to tumor cells, thus favoring the tendency of cancer cells to track along nerves (Shurin et al., 2020; Zhang et al., 2021). Intriguingly, innervated niche-tumor interactions are not limited to the above but extend to altered DNA damage and transcription in cancer cells since the neuronal Soma is in close proximity to the TME (Hara et al., 2011; Zahalka and Frenette, 2020). In addition, multiple growth factors secreted by tumors support axonogenesis, neural reprogramming and neurogenesis directed to the neoplastic front, which in turn initiates bidirectional communication between the tumor and innervated niche (Amit et al., 2016). Exploring the molecular basis of the bidirectional interplay between the tumor and innervated niche holds promise as an entry point for expanding new antitumor strategies (Figure 1).

### The innervated niche orchestrates tumor progression

Angiogenesis is the process of forming new blood vessels from existing vasculature and is necessary for tumor growth and metastasis (Viallard and Larrivée, 2017; Nowak-Sliwinska et al., 2018). Nerves share some profound similarities with vascular networks (mainly arterioles and capillaries), including the same axon guidance molecules (Carmeliet and Tessier-Lavigne, 2005). Four major growth cones sensed-neuronal guidance factors with the ability to regulate developmental and tumor angiogenesis have been identified, including ephrins, netrins, slits, and semaphorins (Eichmann et al., 2005; Klagsbrun and Eichmann, 2005; Larrivée et al., 2009). Akin to the vascular system, lymphangiogenesis and lymphatic remodeling in tumors, which is correlated with tumor aggressiveness, neural regulation is partly dependent on the sympathetic nervous system signaling (Raju et al., 2007; Cao et al., 2013; Le et al., 2013). In fact, sympathetic denervation has been shown to decrease lymphatic vessel formation, similar to the regulation of angiogenesis by the sympathetic nervous system (Raju et al., 2007).

Autonomic innervation directly influences immune cell recruitment and fate in the TME for immunosuppressive (Nance and Sanders, 2007; Li et al., 2022). For example, activated β-adrenergic signaling blocks CD8^+^ T-cell metabolic reprogramming to suppress immune function, thereby facilitating immune escape of tumor cells (Qiao et al., 2019; Lei et al., 2020; Goliwas et al., 2021). β-adrenergic signaling to immune cells subsequently reduces glucose metabolism by inhibiting the expression of the glucose transporter GLUT1, maintaining CD8^+^ T-cells in an anergic state. Additionally, although immune-responsive T helper one cells (TH1 cells) can be recruited to tumors, levels of activated TH1 cells are lower in highly innervated tumors such as in prostate cancer (Bronte et al., 2005; Salmon et al., 2019). In fact, TH1 cells are often prevented from reaching tumors as adrenergic nerves tightly control the efferent lymphatic channels responsible for lymphocyte export, trapping these cells in nearby lymph nodes (Nakai et al., 2014; Suzuki et al., 2016). Adrenergic nerves also increase the generation of immunosuppressive myeloid-derived suppressor cells (MDSCs) from both murine and human peripheral blood cells (Mohammadpour et al., 2019).

Targeted blockade of sympathetic nervous system signals from hepatic stellate cells may be useful in constraining the fibrogenic response to liver injury because sympathetic neurotransmitters directly modulate the phenotype of hepatic stellate cells and induce collagen gene expression (Oben et al., 2003; Oben et al., 2004). Collagens, an important extracellular matrix (ECM) component, are essential for cancer dissemination (Martins Cavaco et al., 2020). Similarly, sympathetic neurotransmitters in the stroma facilitate breast tumor growth by regulating the tumor extracellular matrix, specifically collagen remodeling, whereas norepinephrine depletion inhibits this process (Szpunar et al., 2013). Matrix metalloproteinases (MMPs) are capable of degrading ECM, further facilitating local invasion and dissemination (Kessenbrock et al., 2010). In an orthotopic mouse model of pancreatic cancer, stress-induced neural activation activates adrenergic signaling to increase the MMP expression compartment more than 100-fold and further increase metastasis to the normal adjacent pancreas, while inhibiting the β-adrenergic receptor with propranolol suppresses this phenotype (Kim-Fuchs et al., 2014). In addition, tumor cells exist in the perineurium space, a phenomenon known as perineural invasion, suggesting that the nerve itself provides a structural conduit for tumor cell migration, which is related to tumor metastasis (Marchesi et al., 2010; Jurcak et al., 2019).

### Tumors drive innervated niche alterations

Cancer-associated axonogenesis (the enlargement of nerves or increased nerve density) is a biological phenomenon described from the observation that murine dorsal root ganglia form neurite outgrowths toward prostate cancer cells (Ayala et al., 2001; Ayala et al., 2008). Multiple lines of evidence indicate that tumors secrete multiple neurogenic factors, axon-guidance molecules, and extracellular vesicles to promote axonogenesis (Pundavela et al., 2014; Han et al., 2021; Silverman et al., 2021). For instance, the precursor of nerve growth factor (proNGF) is overexpressed in prostate cancer and involved in the ability of prostate cancer cells to induce axonogenesis (Pundavela et al., 2014). Vascular endothelial growth factor (VEGF), as well as nerve growth factor (NGF), are involved in the axonogenesis of breast cancer (Han et al., 2021). Moreover, tumor-released exosomes contain EphrinB1, which potentiates the induction of PC12 neurite outgrowth (Madeo et al., 2018). On the other hand, Ayala *et al.* first described cancer-related neurogenesis and its putative regulatory mechanism (Ayala et al., 2008). Consistently, a subsequent study confirmed that semaphorin 4F (S4F) has roles in embryologic axon guidance and is involved in cancer-induced neurogenesis (Ding et al., 2013). Lu *et al.* also verified that a fraction of cancer stem cells (CSCs) from patients with gastric carcinoma and colorectal carcinoma were able to generate neurons, including sympathetic and parasympathetic neurons involved in tumor neurogenesis and tumor growth (Lu et al., 2017). Knockdown of the neuron-generating capacity of these stem cells by MAP2 inhibited tumor xenograft growth, thereby underscoring the importance of these *de novo* nerves in cancer progression. Furthermore, neural progenitor cells expressing the neural stem cell marker doublecortin (DCX+) initiate neurogenesis in prostate tumors (Dyachuk et al., 2014; Mauffrey et al., 2019). In this regard, neural progenitor cells of the central nervous system migrate via the bloodstream from neurogenic regions of the brain’s subventricular zone into tumorous and metastatic niches, differentiating into noradrenergic phenotypes, namely, mature neuronal phenotypes. In addition to studying the role of tumors in neurogenesis, the concept that tumors stimulate neural reprogramming has been proposed. In addition, Amit *et al.* expounded on the above deduction, demonstrating that tumor-associated neurons are reprogrammed toward an adrenergic phenotype (Amit et al., 2020). Specifically, the neurons innervating p53-deficient tumors arise from the transdifferentiation of trigeminal sensory fibers to adrenergic nerve fibers. This transdifferentiation corresponds to increased expression of neuronal reprogramming transcription factors, including POU5F1, KLF4, and ASCL1, which are thought to be targets of EV-delivered miR-34a (Hunt and Amit, 2020).

## Autophagy in the innervated niche regulates tumor progression

**FIGURE 2:**
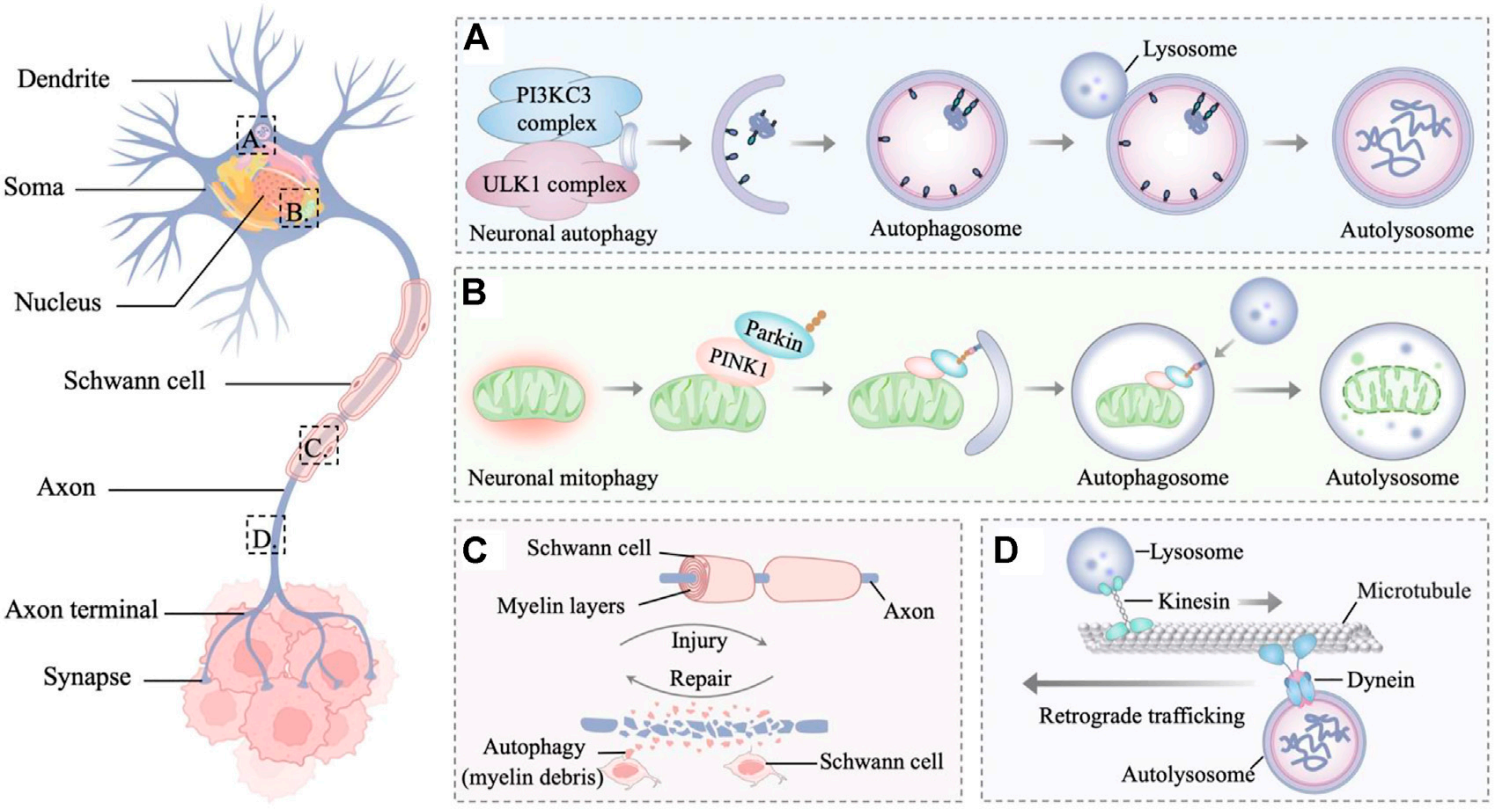
Autophagy in the innervated niche. **(A)** Autophagy is a degradative process in neurons, which involves the sequestration of target proteins and organelles in autophagosomes and subsequent delivery of the cargo to lysosomes for degradation. **(B)** PINK1 accumulates on the outer membrane of damaged mitochondria and recruits parkin to ubiquitinate outer mitochondrial membrane proteins, leading to the mitophagy of damaged mitochondria. **(C)** Autophagy is the main mechanism for myelin debris clearance in Schwann cells. After nerve injury, autophagy in Schwann cells enhances myelin debris clearance to expedite nerve regeneration. **(D)** Retrograde trafficking of axonal autophagosomes to the Soma is microtubule-dependent and driven by dynein motors. Through retrograde transport, the autophagosomes mature and fuse with lysosomes to form mature autolysosomes that are degraded in the neuronal Soma.

Accumulating lines of evidence indicate that autophagy is relevant to neurodegenerative diseases, revealing the importance of autophagy in the nervous system (Martin et al., 2015; Yin et al., 2017; Corti et al., 2020). In the innervated niche, whether upregulated autophagy is stimulative or destructive for tumor progression remains a considerable point of debate. The stimulative effect of autophagy is partly due to its ability to maintain innervated niche homeostasis and even to promote innervated niche development, thereby accelerating tumor progression (Yazdankhah et al., 2021; Griffey and Yamamoto, 2022). By contrast, dysfunctional autophagy potentially causes axon breakdown, dendritic degradation, somal stress and glial cell death, ultimately leading to the disorder of innervated niche (Wang et al., 2021a). The aberrant innervated niche contributes to modulating many critical processes such as angiogenesis, lymphangiogenesis, extracellular matrix remolding, and immune evasion, which could drive the initiative, development, and progression of neoplastic diseases. With an in-depth understanding of autophagy in the innervated niche networks, antitumor therapeutic strategies have been developed by targeting autophagy-related molecules (Figure 2).

The basic unit of the innervated niche is the neuron, a highly specialized cell with activity-dependent plasticity and high metabolic levels (Luo, 2021). Neurons are morphologically polarized in intracellular compartments and are generally divided into three primary constituent compartments: the Soma, the axon, and the dendrite (Terenzio et al., 2017; Holt et al., 2019). The axon is a long extension from the neuronal Soma, growing from a few micrometers up to many feet for conducting information to the nerve terminal (Debanne et al., 2011). While dendrites are typically much shorter, they can build elaborate networks that integrate and process information (Tavosanis, 2021). Cell compartmentalization, long-distance transport, activity-dependent plasticity, and high levels of metabolic stress bring about specific adaptations of neuronal autophagy pathways (Nikoletopoulou and Tavernarakis, 2018; Stavoe and Holzbaur, 2019). In addition, cells that supporting glia in the innervated niche include Schwann cells and satellite glial cells, which provide nutrients and structural support to neurons (Jessen et al., 2015; Hanani and Spray, 2020). As glial stress may lead to neuronal pathology, dysfunction within the innervated niche is partly associated with glial stress, whereas autophagy in glial cells plays a regulatory role in this process (Aschner et al., 1999; Jessen, 2004; Jessen and Mirsky, 2016). In the following sections, the dynamic interactions between autophagy and different components of the innervated niche will be described in more detail to analyze how autophagy in the innervated niche protects against tumors from different perspectives.

### Somal stress inhibits tumor progression

Less is known about macroautophagy in neuronal Soma than for axons and dendrites. Indeed, in primary dorsal root ganglion (DRG) neurons, autophagosome biogenesis is enriched distally and infrequently forms in Soma and dendrites (Maday and Holzbaur, 2014). Maday *et al.* found that occasionally autophagosomes confined within the Soma are less mobile and tend to cluster (Maday and Holzbaur, 2016). Autophagosomes from axons enter the Soma and stay in the dendritic domain, which is associated with the accumulation of proteolytically active lysosomes in the Soma. As for neuronal mitophagy, there are still some conflicting views about whether it occurs in the Soma. To combat oxidative stress, the PINK1-Parkin-mediated mitophagy pathway is required in local axons to provide rapid neuroprotection without a requirement for retrograde transport to the Soma (Ashrafi et al., 2014). Conversely, the selective release of the mitochondrial anchoring protein syntaphilin from axonal stressed mitochondria enhances their retrograde transport toward the Soma from the axon (Lin et al., 2017a; Lin et al., 2017b). Cai *et al.* found the dynamic formation and elimination of Parkin- and LC3-ring-like structures surrounding depolarized mitochondria in the Soma through the autophagy-lysosomal pathway (Cai et al., 2012). Sung *et al.* provided additional convincing evidence in the *Drosophila* nervous system that the Soma is the focal location of PINK1/Parkin-dependent mitophagy in neurons (Sung et al., 2016). Furthermore, an *in vivo* study from a mouse model demonstrated that expressing a mitophagy reporter (mito-QC) also supports a degradation pathway in neuronal Soma (McWilliams et al., 2016). The mitophagy-deficient Soma can impair the innervated niche, and the impaired cancer-nerve crosstalk consequently suppresses tumor development (Boilly et al., 2017; Wang et al., 2021b). In summary, intervention strategies targeting key molecules of the mitophagy pathway in the innervated niche may represent promising approaches to inhibit tumor progression.

### Axonal breakdown suppresses tumor development

Landmark studies on the ultrastructural analysis of cultured neuronal axons revealed the presence of autophagic vesicles in distal growth cones (Yamada et al., 1971; Bunge, 1973). Subsequently, Hollenbeck observed real-time autophagosome biogenesis and dynamics showing that acidified vesicles formed in the local axon and were transported retrogradely toward the Soma (Hollenbeck, 1993). This work was consistent with observations of LC3-positive organelles forming constitutively at the axonal tip (Lee et al., 2011). This powerful retrograde transport capability of autophagosomes has also been corroborated *in vivo* in model organisms (Stavoe et al., 2016; Hill et al., 2019). In *Caenorhabditis elegans*, the synaptic vesicle kinesin (KIF1A/UNC-104) localizes autophagosome biogenesis near synapses in axons by regulating the transport of the integral membrane autophagy protein ATG-9 (Stavoe et al., 2016). In *Drosophila*, the scaffold protein CKA facilitates axonal transport of autophagosomes in a PP2A-dependent fashion (Neisch et al., 2017). Furthermore, a specific role of synaptojanin 1 (SynJ1) in autophagosomal and endosomal trafficking was demonstrated in a SynJ1-deficient zebrafish mutant (George et al., 2016). In neuronal axons, the activation of autophagy promotes axon regeneration that lacks regrowth capacity, providing promising support for maintaining innervated niche homeostasis (Ko et al., 2020).

Many autophagosomes that form at the distal axons need to be delivered to somatic cells, although partial local autophagic degradation has also been shown in axons (Farfel-Becker et al., 2019; Kuijpers et al., 2021). This is because the hydrolase-enriched lysosomes are viewed as essential for degradation and predominantly localized in neuronal somas (Sharoar et al., 2021). As autophagosomes move distally to proximally along the axon, autophagosomes mature and gradually acidify to form autolysosomal compartments that more efficiently catalyze the degradation of enveloped cargoes (Maday and Holzbaur, 2012). This centripetal movement of the axonal autophagosome is a microtubule-dependent transport along the axon, driven by kinesin and dynein motors (Maday et al., 2012; Cheng et al., 2015). The late endosome-loaded dynein-snapin complex mediates retrograde transport after autophagosomes fuse with late endosome into amphisomes to maintain efficient autophagy (Tammineni et al., 2017). Consistent with this, deficiency of dynein-snapin coupling impairs autophagosome trafficking, resulting in autophagic stress in neuronal axons (Cheng et al., 2015). Furthermore, the motor scaffolding protein JIP1 regulates the processive dynein-driven transport of autophagosomes in neurons (Fu et al., 2014). The autophagosome adaptor LC3 directly binds JIP1 through a conserved LIR motif, while JIP1 directly binds to the dynactin activator dynactin and activates kinesin one in a phosphorylation-dependent manner. Additionally, the phenomenon of disrupting lysosomal trafficking to induce axonal autophagic stress suggests the importance of local degradation of axons (Farfel-Becker et al., 2020). Soma-derived degradative lysosomes rapidly influx into distal axons, targeting autophagosomes and SNCA/α-synuclein cargos for local degradation. Taken together, axonal autophagy is critical for reducing stress in the innervated niche; thus, targeted disruption of axonal autophagy, such as microtubule-dependent autophagosome transport, can specifically suppress tumor development by impairing the innervated niche (Zahalka and Frenette, 2020; Silverman et al., 2021).

### Dendritic degradation regulates tumor growth

Studies have shown that autophagy regulation also exerts an important function in neuronal dendrites. Increased dendritic spine density with reduced developmental spine pruning correlates with hyperactivated mTOR and impaired autophagy (Tang et al., 2014). In cultured rat neurons, upon stimulation with low-dose NMDA, dendritic spines displayed an increase in GFP-LC3 puncta with a time course coincident with Akt and mammalian target of rapamycin (mTOR) dephosphorylation (Shehata et al., 2012). Kulkarni *et al.* found that synaptic activity increases the local and reversible movement of degraded autophagosomes in dendrites instead of axons (Kulkarni et al., 2021). An intriguing finding is that autophagy regulates neuronal dendritic branching to promote dendritic diversification (Clark et al., 2018). Autophagy induction partially rescued the dendritic atrophy defect in a polyglutamine toxicity model (Clark et al., 2018). The paradox is the transcriptional control of basal autophagy in dendritic terminal branching. Knockdown of autophagy-related genes (Atg) reduced dendritic arbor growth and terminal branching in multidendritic sensory neurons in *Drosophila*, whereas excessive autophagy led to dramatic reductions in dendritic complexity (Clark et al., 2018).

Additionally, increasing evidence suggests that autophagy modulates dendritic degeneration. In conditional knockout mice, Atg7-deficient neurons exhibited early dendritic dystrophy and the formation of dendritic ubiquitinated inclusions (Friedman et al., 2012). As a stress-response protein, NAD synthase nicotinamide mononucleotide adenylyltransferase (NMNAT) displays chaperone function in neuronal maintenance and protection (Zhai et al., 2008). Neuronal NMNAT loss triggers the exposure of the autophagic signal phosphatidylserine and leads to autophagy-dependent dendritic degeneration (Ji et al., 2022). Under hypoxic stress, genetically blocking autophagy suppresses hypoxia-induced dendrite degeneration of NMNAT heterozygous mutants, suggesting a self-destructive role for autophagy in this context (Wen et al., 2013). Therefore, the homeostatic autophagy within the innervated niche needs to be tightly regulated to balance dendrite branching and growth under native or cellular stress conditions. Accordingly, autophagy mediates dendritic degradation in the innervated niche, which may represent an effective strategy for inhibiting tumor growth.

### Glial cell death disrupts the tumor microenvironment

Similar to previous studies in neurons, glial autophagy in the innervated niche has mainly been studied as a stress reactivity pathway (Belgrad et al., 2020). As the cells responsible for axon guidance, structural support and secretion of growth factors, Schwann cells and their homeostasis have received much attention (Nazareth et al., 2021). Excessive autophagy induced by oxidative stress leads to the death of Schwann cells, whereas docosahexaenoic acid (DHA) attenuates this phenomenon (Tatsumi et al., 2022). In contrast, epothilone B (EpoB) promotes axonal regeneration following peripheral nerve injury by promoting PI3K/Akt signaling-mediated autophagy in Schwann cells and enhancing migration (Zhou et al., 2020). Moreover, autophagy in Schwann cells, which can be activated by nerve growth factor, is crucial for myelin debris clearance to expedite nerve regeneration following peripheral nerve injury (Li et al., 2020). Correspondingly, deletion of calcineurin reduces autophagic flux in Schwann cells and thus delays myelin degradation (Reed et al., 2020). Lutz *et al.* suggest that Schwann cells collaborate with hematogenous macrophages to clear myelin debris through TAM receptor-mediated phagocytosis and autophagy in a mouse nerve injury model (Brosius Lutz et al., 2017). In the TME, Schwann cells are extensively distributed and could serve as a prognostic factor for poor survival of patients with PDAC (Su et al., 2020a). Further research found that autophagic Schwann cells exert a “nerve-repair like effect” to promote perineural invasion in pancreatic cancer (Zhang et al., 2022a). These results indicate that autophagy in Schwann cells is an important factor for maintaining the innervated niche and suggests targeting Schwann cells with autophagy inhibitors as a possible tactic for eliminating tumors (Sun et al., 2022).

## Drug repurposing for cancer therapy: Targeting autophagy in the innervated niche

Ever-present obstacles to the discovery of new drugs, particularly in the context of cancer therapy, have necessitated the development of alternative strategies for drug repurposing (Zhang et al., 2020). Drug repurposing, also known as drug repositioning, refers to the concept of the alternative application of a drug developed for another indication in novel therapeutic indications (Würth et al., 2016). Antimalarial agents such as artemisinin are typical examples of drug repurposing that affect autophagy regulation for therapeutic use against cancer (Efferth, 2017; Wong et al., 2017). The advantages of drug repurposing are that important drug characteristics have already been established in clinical databases (including pharmacokinetics, pharmacodynamics, toxicity and efficacy), reducing the valley between drug discovery and commercial availability (Parvathaneni et al., 2019).

**FIGURE 3:**
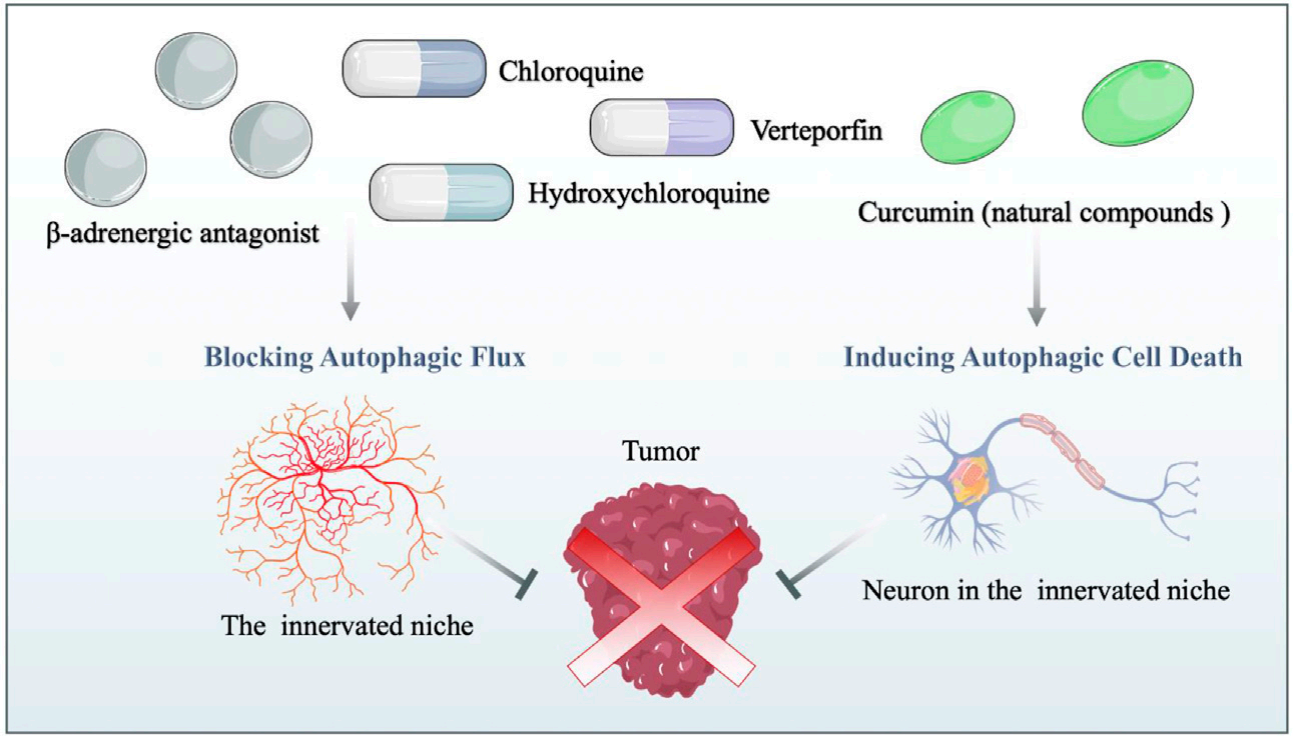
Drug repurposing for cancer therapy based on the autophagy-mediated innervated niche. Drug candidates disrupt the innervated niche by blocking autophagic flux and inducing autophagic cell death, thereby inhibiting tumor progression. β-adrenergic antagonists: candidates for targeting the innervated niche.

As mentioned above, the innervated niche supports neoplastic signaling pathways and contributes directly to cancer microenvironment development. Moreover, autophagy dynamically modulates components of the innervated niche; thus, targeting autophagy can disrupt the homeostasis of the innervated niche in multiple ways. In this context, disordering the innervated niche by regulating autophagy emerges as a novel therapeutic opportunity for cancer treatment. For new pharmaceutical development with rapid clinical translation, repurposing existing drugs based on the autophagy-mediated innervated niche could be cost-effective and advantageous in human neoplastic disease therapy (Figure 3). Herein, this chapter outlines drugs repurposing for cancer therapy from the viewpoint of the autophagy-mediated innervated niche (Table 1).

**TABLE 1:** The summary of drug candidates in new antitumor application.

Drug	Conventional indication	Molecular targets	Autophagy modulating mechanism	Targeting cancer	Clinical trials
Propranolol	Hypertension; angina pectoris	β-adrenergic receptor	Blocking late stage of autophagy	Breast cancer Montoya et al. (2019); gastric cancer Koh et al. (2021)	NCT02596867; NCT03245554
Carvedilol	Heart failure	β-adrenergic receptor	Increasing the lysosomal pH; accumulating immature autophagosomes	Breast cancer Gillis et al. (2021)	NCT02177175
Chloroquine	Malaria; rheumatoid arthritis	Lysosome	Blocking autophagosome-lysosome fusion	Pancreatic cancer Nakano et al. (2020); breast cancer (Maycotte et al., 2012)	NCT01777477; NCT01446016
Hydroxychloroquine	Malaria; autoimmune disease	Lysosome	Blocking autophagosome-lysosome fusion	Pancreatic cancer Karasic et al. (2019); colorectal cancer Yao et al. (2016)	NCT01273805; NCT01006369; NCT00726596
Verteporfin	choroidal neovascularization	p62	Blocking autophagic flux	Breast cancer Wei and Li, (2020)	NCT02872064
Curcumin	Inflammatory diseases	Nrf2; AKT/mTOR	Induce autophagic cell death	Breast cancer Mir et al. (2018); colorectal cancer Weng and Goel, (2022); prostate cancer Mir et al. (2018)	NCT01042938; NCT02439385; NCT03211104

The β-adrenergic receptor is widely expressed in various human tissues and organs, mediating essential body functions (Molinoff, 1984). Previous studies have shown that the influence of the innervated niche on cancer initiation, progression, and metastasis is primarily mediated through β-adrenergic receptor signaling (Mravec et al., 2020; Kamiya et al., 2021; Yonekura et al., 2022). Specifically, aberrant activation of β-adrenergic receptor signaling is associated with multiple hallmarks of cancer, including cell proliferation, cell death resistance, cancer invasion and metastasis, and angiogenesis (Pérez-Sayáns et al., 2010; Tang et al., 2013). Given that β-adrenergic signaling is considered an important neural regulation pathway in the innervated niche, which is highly involved in regulating tumors by activating different downstream signal transduction pathways, repurposing β-adrenergic receptor antagonists as antitumor agents has received consideration (Velmurugan et al., 2019). β-adrenergic antagonists (β-blockers) can be categorized into three generations according to their pharmacological properties: first-generation β-blockers such as propranolol are non-selective, blocking both β1-and β2-receptors; second-generation β-blockers such as metoprolol are more selective for β1-receptors; and third-generation β-blockers such as carvedilol are highly selective drugs for β1-receptors (do Vale et al., 2019). Oral propranolol has been used to treat complex infantile hemangiomas, and preclinical experiments have demonstrated the inhibitory effect of propranolol in a variety of cancers by mechanisms that do not wholly involve adrenergic signaling, supporting propranolol as a promising candidate for drug repurposing (Léauté-Labrèze et al., 2015; Montoya et al., 2019; Koh et al., 2021). Repurposing propranolol with a combination of metformin as an adjuvant strategy for colorectal and triple-negative breast cancers has been proposed by Anselmino *et al.* (Anselmino et al., 2021). Moreover, cancer cells are particularly sensitive to propranolol in the condition of enhanced autophagic flux, related to the late stage of autophagy blocked by propranolol (Brohée et al., 2018). In addition, carvedilol blocks neural regulation of breast cancer progression by reducing SNS activation, which is associated with reduced breast cancer mortality in patients (Gillis et al., 2021). Carvedilol increases the number of GFP-LC3-containing puncta and LC3B-II levels in a time-dependent manner, while adding chloroquine fails to enhance the increased autophagic marker levels (Meng et al., 2018). Unexpectedly, rather than impairing the fusion of autophagosomes with lysosomes directly, carvedilol inhibits autophagic flux by increasing lysosome pH and the accumulation of immature autophagosomes characterized by lower acidity and hydrolytic properties (Meng et al., 2018; Rivero et al., 2021; Lu et al., 2022). Based on the above, one possible area for repurposing β-adrenergic antagonists in cancer therapy is the use of neuromodulators targeting autophagy in the innerved niche. However, evidence to support the direct connection between the innerved niche and autophagy mediated by β-adrenergic antagonists is still lacking. That said, these existing studies on β-adrenergic antagonists on autophagy, the innerved niche and the whole organism at least indicate their potential repurposing in future cancer therapy.

### Chloroquine, hydroxychloroquine and verteporfin: Clinically available autophagy inhibitors

With an original indication to prevent or cure malaria, chloroquine and hydroxychloroquine have been successfully used to treat several infectious diseases (D'Alessandro et al., 2020), rheumatology (Schrezenmeier and Dörner, 2020) and immunological diseases (Ben-Zvi et al., 2012). Due to their remarkable ability to block autophagy, chloroquine and hydroxychloroquine have also been widely reported as potential anticancer drugs (Ferreira et al., 2021). Mechanistically, they are inhibitors of lysosomal acidification that block autophagosome-lysosome fusion in the autophagic late phase, resulting in the accumulation of acidic vesicular organelles in the cytoplasm (Pasquier, 2016). In innervated niches, autophagic stress caused by dysfunction of autophagosome-lysosome fusion is lethal to highly differentiated neurons (Farfel-Becker et al., 2020). It is an indisputable fact that deficits in neuronal lysosome function and dysfunction of autophagy result in neurodegeneration (Roney et al., 2022). Based on this, we hypothesize that blockage of the autophagy flux by chloroquine and hydroxychloroquine not only diminishes the positive effect in maintaining the innervated niche homeostasis but even leads to lethal autophagic stress. Indeed, in the neuronal cell model (M1C cells), chloroquine damages neuronal cells accompanied by a significant increase in the accumulation of tau protein, suggesting autophagy lysosomal system disturbances cause neuronal stress by perturbing the degradation mechanisms of tau protein (Hamano et al., 2021). Furthermore, chloroquine directly excites neurons in a receptor-dependent manner (Liu et al., 2009). Essentially, chloroquine and hydroxychloroquine are neuromyotoxins that affect nerves, cardiac and skeletal muscles (Estes et al., 1987; Stein et al., 2000). Morphologic changes in human peripheral nerves under chloroquine treatment confirm its neuromyotoxicity. Accordingly, it is inferred that chloroquine and hydroxychloroquine also directly damage the innervated niche through their own toxicity, thereby limiting the development of cancer. Taken together, the use of chloroquine and hydroxychloroquine as repurposed drugs for cancer, either by directly damaging the innervated niche or indirectly blocking autophagic effects in the innervated niche appears beneficial.

Verteporfin is an FDA-approved photosensitizer clinically used in photodynamic therapy for the treatment of choroidal neovascularization, especially in age-related macular degeneration (Brodowska et al., 2014). Based on the present *in vivo* evidence, verteporfin may be repurposed as a promising chemotherapeutic and adjuvant drug for cancer therapy (Feng et al., 2016; Liang et al., 2020). While verteporfin effectively interferes with the YAP/TAZ interaction with TEADs, other effects of verteporfin have been described, such as inhibiting the formation of autophagosomes (Gibault et al., 2016). Autophagy initiates at the pre-autophagosomal structure by forming a phagophore, which gradually elongates and closes to form a double-membrane autophagosome containing cytoplasmic cargo (Dikic and Elazar, 2018). Although both verteporfin and chloroquine are autophagy inhibitors, unlike chloroquine, verteporfin blocks autophagic flux prior to autophagolysosome formation by blocking p62 oligomerization (Saini et al., 2021). The importance of autophagosomes in neuronal homeostasis has been demonstrated, partly because loss of autophagosome biogenesis results in protein aggregation (Menzies et al., 2017; Stavoe and Holzbaur, 2019). In addition to neurons, activation of autophagy in glial cells promotes perineural invasion in pancreatic cancer, re-emphasizing the importance of inhibiting the autophagy pathway (Zhang et al., 2022a). Therefore, verteporfin-mediated impaired autophagy conceptually inhibits innervated tumor development, thus making verteporfin promising for repurposing as an antitumor drug. Disappointedly, there is no conclusive evidence to directly show that verteporfin exerts antitumor effects by targeting the autophagy-mediated innervated niche, and further studies are required to clarify this issue.

### Curcumin: Typical natural compounds

While several synthetic autophagy modulators have been identified as promising cancer therapy candidates, natural compounds and their derivatives have also attracted significant attention for use as autophagy modulators in cancer treatment with minimal side effects (Moosavi et al., 2018; Deng et al., 2019; Luo et al., 2019; Vidoni et al., 2020). Curcumin, one of the typical natural compounds used in traditional Chinese medicine, has been extensively investigated from the pharmacological point of view (Priyadarsini, 2014). Increasing evidence indicates that curcumin, as a naturally occurring autophagy modulator, can induce autophagic cell death and autophagy-mediated apoptosis, which can be reversed by inhibiting autophagy (Seo et al., 2018; Chen et al., 2019b; Shakeri et al., 2019; Rainey et al., 2020). Autophagy-related cell death leads directly to the death of neuronal and glial cells, which contributes to the neuropathy of the innervated niche (Ngwenya and Danzer, 2018; Stavoe and Holzbaur, 2019). Hence, drug repurposing of curcumin conceptually shows therapeutic promise for highly innervated tumors. In addition, other signaling pathways, such as the Wnt/beta-catenin pathway and NF-kappaB pathway, have been shown to be modulated by curcumin, resulting in cancer inhibition (Kunnumakkara et al., 2017; Weng and Goel, 2022). Researchers have recommended the co-administration of curcumin with chemotherapeutic drugs to enhance synergistic anticancer efficacy while minimizing undesirable toxicity (Howells et al., 2019; Zheng et al., 2021). Interestingly, curcumin has been shown to activate Nrf2 and exert neuroprotective effects against oxidative stress in neurological disorders (Dong et al., 2018; Shahcheraghi et al., 2021). Many natural compounds derived from natural sources have been shown to activate adaptive stress responses at low doses, while acute responses such as cell death are activated at high doses (Mattson and Cheng, 2006; Mattson, 2008). Additional investigations are needed to determine the most appropriate dose fractionation schedule for lethal autophagy and local innervated niche damage with minimal toxicity. Enhanced bioavailability and tissue targeting of natural compounds by nanoengineering promises to bring natural products similar to curcumin to the forefront of drugs repurposing for cancer therapy in the near future (Mahjoob and Stochaj, 2021; Xu et al., 2021).

## Future challenges and conclusion

Emerging research in cancer neuroscience is devoted to revealing how the innervated niche synergistically contributes to the initiation, metastasis and therapeutic resistance of cancers. However, much remains to be figured out about the pathophysiology of cancer in the innervated niche, which needs to be explored with the help of the powerful tools available to modern cancer neuroscience (Jiang et al., 2022). In addition, interactions between different malignancies and the innervated niche underscore the co-evolution and individual-specific differences. Therefore, high-throughput single-cell sequencing coupled with novel methods such as lineage analysis will help associate particular cancer phenotypes with the heterogeneous innervated niche (Weaver et al., 2014; Sun et al., 2015). Similar to the indispensable regulation of the innervated niche in tumors, innervation is an inevitable part of the whole organism (Elenkov et al., 2000; Elefteriou, 2018). Accordingly, the systemic effects in treatment should be carefully considered when repurposing drugs based on the innervated niche. Inspired by targeted drug delivery systems in nanomedicine, engineering repurposing drugs with localized and targeted release ability can be one of the entry points (Park, 2014; Srinivasarao and Low, 2017; Jiang et al., 2020; Ming et al., 2022). There is an urgent need for integrated interdisciplinary research and collaboration between the disciplines of pharmacy, neuroscience and cancer research to achieve more precise applications.

Current antitumor drugs are highly cytotoxic and focus on rapidly eradicating dividing cancer cells (Slade, 2020). The concept of inducing a surge in cell death seems plausible since the induction of programmed cell death through apoptosis, ferroptosis, pyroptosis, *etc.*, has been extensively studied (Shi et al., 2017; Liang et al., 2019; Su et al., 2020b; Carneiro and El-Deiry, 2020; Zhang et al., 2022b). Nevertheless, drug repurposing aimed at modulating autophagy in the innervated niche differs from traditional strategies of inducing cell death. Drugs targeting the autophagy-mediated innervated niche modulate multiple hallmarks of cancer to kill cancer cells indirectly, suggesting it may be used as a combination strategy to cascade-amplify tumor inhibition (Silverman et al., 2021). Challenges and opportunities exist in accurately identifying patients most likely to benefit from this approach and maximizing the effect of repurposed drugs through single-agent or combination therapeutic strategies. In addition, existing studies indicate that autophagy plays a dynamic and complex role in the innervated niche, partly explaining the duplicity of autophagy in cancer. This again highlights the requirement for additional studies to understand the context-dependent role of autophagy in the innervated niche. Evidence to support the effect of the autophagy-mediated innervated niche on drug repurposing is currently lacking but is conceptually plausible. Consequently, *in vivo* experiments are urgently needed to further evaluate the clinical applications of the autophagy-mediated innervated niche in drug repurposing.

In summary, it is now understood that the innervated niche is a complicated and heterogeneous niche that can affect cancer development. It has great potential as a therapeutic target which could ultimately provide new opportunities for improving outcomes of many difficult-to-treat malignancies. Therefore, targeting the innervated niche through the autophagy pathway has emerged as a novel window for drug repurposing. Drug repurposing based on autophagy in the innervated niche should be considered a complement to the new paradigm of precision medicine in the future, which may lead to a higher success rate in clinical application.
